# Proof of Concept for an Intracochlear Acoustic Receiver for Use in Acute Large Animal Experiments

**DOI:** 10.3390/s18103565

**Published:** 2018-10-21

**Authors:** Flurin Pfiffner, Lukas Prochazka, Ivo Dobrev, Karina Klein, Patrizia Sulser, Dominik Péus, Jae Hoon Sim, Adrian Dalbert, Christof Röösli, Dominik Obrist, Alexander Huber

**Affiliations:** 1 Department of Otorhinolaryngology, Head and Neck Surgery, University Hospital Zurich, University of Zurich, 8091 Zurich, Switzerland; lukas.prochazka@usz.ch (L.P.); ivo.dobrev@usz.ch (I.D.); patrizia.sulser@kispi.uzh.ch (P.S.); dompeus@gmail.com (D.P.); jaehoon.sim@usz.ch (J.H.S.); adrian.dalbert@usz.ch (A.D.); christof.roeoesli@usz.ch (C.R.); alex.huber@usz.ch (A.H.); 2 Vetsuisse Faculty, University of Zurich, 8057 Zurich, Switzerland; kklein@vetclinics.uzh.ch; 3 ARTORG Center, University of Bern, 3010 Bern, Switzerland; dominik.obrist@artorg.unibe.ch

**Keywords:** acoustic receiver, auditory system, biomedical measurement, BioMEMS, cochlea, ear, hydrophone, implantable microphone, implantable sensor, intracochlear sound pressure, sheep, totally implantable cochlear implant

## Abstract

(1) Background: The measurement of intracochlear sound pressure (ICSP) is relevant to obtain better understanding of the biomechanics of hearing. The goal of this work was a proof of concept of a partially implantable intracochlear acoustic receiver (ICAR) fulfilling all requirements for acute ICSP measurements in a large animal. The ICAR was designed not only to be used in chronic animal experiments but also as a microphone for totally implantable cochlear implants (TICI). (2) Methods: The ICAR concept was based on a commercial MEMS condenser microphone customized with a protective diaphragm that provided a seal and optimized geometry for accessing the cochlea. The ICAR was validated under laboratory conditions and using in-vivo experiments in sheep. (3) Results: For the first time acute ICSP measurements were successfully performed in a live specimen that is representative of the anatomy and physiology of the human. Data obtained are in agreement with published data from cadavers. The surgeons reported high levels of ease of use and satisfaction with the system design. (4) Conclusions: Our results confirm that the developed ICAR can be used to measure ICSP in acute experiments. The next generation of the ICAR will be used in chronic sheep experiments and in TICI.

## 1. Introduction

During the hearing process, airborne sound is transmitted and transformed through the outer and middle ear into sound pressure in the fluid filled inner ear (cochlea). Acoustic input to the cochlea induces intracochlear sound pressure (ICSP) variations causing basilar membrane motion and excitation of sensorial structures. Investigation of ICSP is an important objective method that has been used to evaluate the biomechanical process of hearing.

Intracochlear acoustic receivers (ICAR) have been used to measure the sound pressure in the cochlea. They are miniature hydrophones that can operate in a liquid medium such as the cochlear fluid (perilymph). They must be sufficiently small to accommodate the anatomical dimensions of the inner ear but at the same time provide high sensing performance for reliable ICSP measurements.

So far, ICARs based on different transduction mechanisms have been developed, including fiber-optic [1,2,3], piezoelectric [4], piezoresistive [5], capacitive [6], strain gauges [7], and servo micropipette systems [8]. They have mainly been used for experiments in the cadavers of small animals and in human temporal bones.

To gain further insight into the biomechanics of hearing, in vivo ICSP measurements in animals (acute and chronic) are essential in order to exclude the influence of post-mortem effects. Such effects might include the absence of vital functions (e.g., labyrinth pressure, blood circulation and cellular metabolism, ear muscle function and active amplification processes of the inner ear), and the conservation treatment of cadaver samples (i.e., freezing, thawing, dehydration, and autolysis) [9,10,11].

Compared to experiments on cadaver temporal bones, acute in vivo ICSP measurements are more complex mainly due to the restricted surgical access to the inner ear and the time needed for the intervention. Chronic in vivo animal experiments impose additional requirements on the ICAR design in order to allow preservation of hearing and physiological functions. Such requirements include biocompatibility, long term hermeticity, an implantable data readout system and stable performance over time. None of the existing ICARs that have been reported in the literature can be used as an implantable sensor for chronic ICSP experiments.

Acute ICSP measurements in vivo have only been performed in small anesthetized animals such as cats [12], guinea pigs [13] and chinchillas [14]. These animals have significantly different anatomy and physiology compared to humans, thus allowing only limited conclusions regarding the biomechanics of human hearing to be drawn. In contrast, studies have shown that sheep are a valid large animal model for research in otology that can be used as a surgical training model because of the similarity in cochlear access and to validate hearing devices for human use [15,16,17]. The sheep and human cochlea share similarities in size, shape and biomechanical performance (e.g., frequency range of hearing) [18].

In this work, we investigated a partially implantable acoustic receiver fulfilling the requirements for acute ICSP measurements in a live specimen that is representative of the anatomy and physiology of the human. The aims were to (1) validate the ICAR’s performance under laboratory conditions with functional tests; (2) to validate its use for ICSP measurements for the first time in a live specimen that is representative of the anatomy and physiology of the human.

Evaluating the feasibility and ease of use of the ICAR and gaining procedural knowledge from the acute in vivo ICSP measurements in sheep are important milestones towards developing an ICAR system used for chronic animal experiments and as an implantable microphone for totally implantable hearing devices (e.g., cochlear implants [19]).

## 2. Materials and Methods

### 2.1. ICAR Requirements

The most important requirements to be considered for an ICAR to be used for acute ICSP measurements included a form factor suitable for the small anatomical size of the ear structures, sufficient and stable sensing performance during experiments, and simple surgical handling to insert the ICAR into the cochlea.

To measure ICSP, minimally invasive access to the middle ear cavity (in sheep: mean length 13.3 mm, mean height 18.9 mm [20]) and inner ear (internal diameter of the total cochlear basal turn: 4.9 mm, [21]) that preserves all anatomical structures important for sound transmission to the cochlea fluid was required. The restricted space conditions demanded an appropriate ICAR geometry that did not interfere with structures such as the middle ear ossicles, ligaments, and muscles in the middle ear cavity. In addition, an ICAR form factor that allowed a sufficient view for insertion into the cochlea and a small access to the inner ear that minimized the influence of the ICAR on the cochlear fluid dynamics was necessary.

In contrast to experiments on cadavers, acute experiments with anaesthetized animals are more restricted in time and demand a simple, reliable and standard surgical approach for ICAR insertion. Therefore, the ICAR design had to allow for repeated ICAR insertions and fixation by the surgeon without the need for a dedicated holder and associated micromanipulator.

The required sensing performance for acute ICSP measurements was defined as covering the essential hearing range (i.e., between 0.25 and 8 kHz) with a signal-to-noise ratio (SNR) greater than 6 dB for acoustic stimulation above a 90-dB sound pressure level (SPL). Stable sensing performance (within ±3 dB) had to be maintained during the experiments.

Certain requirements such as biocompatibility, hermeticity, low power consumption, and an implantable read out system, as required for chronic tests or for a microphone of a totally implantable cochlear implant, were not evaluated in this phase of ICAR development. Nevertheless, the ICAR concept was designed such that all of these features could be incorporated in a later stage of development.

### 2.2. Sensor Design

Capacitive transduction was used as the sensing principle for the present ICAR. Recent work has shown that micro-electro-mechanical system (MEMS) condenser microphones (CMIC) are the sensor technology that best fulfills the requirements of high sensing performance combined with good system integration capabilities and low power consumption [6]. Therefore, a CMIC was considered to be well suited for potential future use as an inner ear microphone for chronic animal experiments and fully implantable cochlea implant systems.

**Figure 1:**
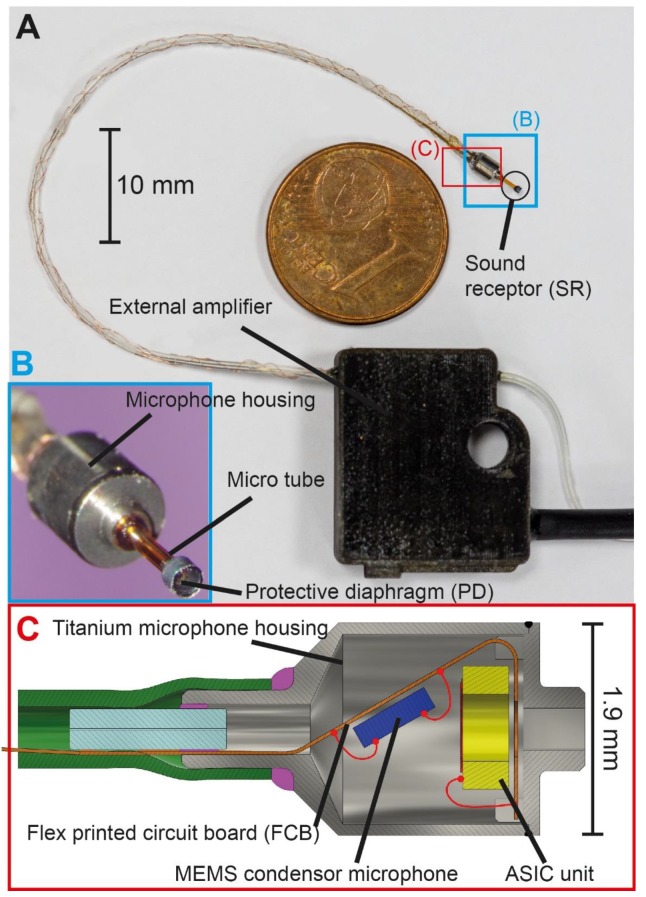
(**A**) Photograph of the completely assembled ICAR, (**B**) Zoomed-in view of the ICAR head, (**C**) Schematic drawing of the microphone housing cross section.

The MEMS CMICs used a 1-mm^2^ large-plate capacitor with a movable membrane of submicron thickness (diaphragm) as the sound pressure sensing element. These acoustic receivers were designed for sensing in air (or other gases) and cannot operate in a liquid medium mainly due to electrical insulation and a non-hermetic diaphragm design. Our ICAR concept for fluid immersion included an additional passive protective diaphragm (PD) sealing the MEMS CMIC against the liquid medium on the receiving side (Figure 1B). Vibrations of the PD induced by ICSP were transferred to the diaphragm of the CMIC by pressure fluctuations in the air-filled volume enclosed by the two deflecting elements. The MEMS CMIC itself was too large to fit inside the cochlea and was therefore situated in the surgical access cavity to the inner ear. Only a micro tube of several millimeters in length, sealed by the PD at the front end and interconnected with the MEMS CMIC at the other end, was inserted into the cochlear duct (Figure 1).

Existing ICARs that are designed for measurements in cadavers usually require a dedicated micromanipulator or holder for insertion into the inner ear [6]. The study design allowed the surgeon to insert and fix the ICAR using a surgical approach similar to that used for clinical cochlear implants and did not require complex assistive tools. This was achieved by using custom-made packaging for the MEMS CMIC with an optimum form factor and a flexible interface between the microphone and the external amplifier unit (Figure 1).

### 2.3. Sensor Parts

The ICAR developed for the study consisted of three main components: the sound receptor (SR) unit, the custom-made microphone housing, and the external amplifier unit (Figure 1). The micro tube of the SR was formed of polyimide-coated fused silica with an outer diameter of 0.36 mm, a capillary diameter of 0.15 mm, and a length of 2 mm. The front end (part inserted into cochlea fluid) of the tube was sealed with the PD made of a 1-micron thick polyimide film supported by a thin walled (0.075 mm) cylindrical structure of single crystal silicon with an outer diameter of 0.5 mm. Polymer was chosen as the diaphragm material because of the simplicity of fabrication. The MEMS fabrication process on the wafer level was based on standard photolithography and dry anisotropic etching of single crystal silicon.

The microphone housing, shown in Figure 1C, consisted of two micro-machined parts—the base plate and the enclosure—both made of titanium. The base plate held the MEMS CMIC and contained the interfacing port for the SR. The enclosure formed the back cavity of the acoustic receiver and protected the delicate CMIC from humidity and mechanical impact. A small opening within the closed end of the enclosure served as a feed through for the electrical and pneumatic interface required for static pressure equalization (SPEQ). The outer diameter of the housing was 1.9 mm and the overall height was 3.2 mm.

A low-power MEMS CMIC (type ADMP803, InvenSense Inc., San Jose, CA, USA) on the chip level was used for the ICAR. The microphone and the corresponding ASIC unit were electrically and mechanically interfaced using a 30-micron thin flexible printed circuit board (FCB), which allowed bending or even folding of the FCB (bend radius < 0.2 mm). The FCB also included a 100-mm long flex print cable, which simplified the electrical interfacing of the microphone and the amplifier unit. To maintain SPEQ between the environment and the ICAR’s interior, a 1.5-mm long fused silica tube with a capillary diameter of 20 microns was inserted into the pressure port of the enclosure and tightly sealed. This tiny micro tube served as an acoustic throttle with a low cut-off frequency less than 100 Hz. Finally, the base plate and the enclosure were micro laser welded to obtain a robust and gas-tight seal. The microphone housing and the ground plane of the FCB were electrically interconnected to provide electrical shielding of the ICAR.

The external amplifier unit had electrical circuitry that provided the output signal amplification (gain = 50, type OP ADA4004, Analog Devices, Norwood, MA, USA), DC removal, and power supply of the MEMS CMIC (0.9–1.3 V). The housing was made from an acrylic photopolymer designed to simplify the electrical interface between the FCB cable and amplifier unit and to hold and protect the ICAR during handling. A flexible, 100-mm long silicone tube (inner diameter: 0.3 mm, outer diameter: 0.6 mm) interconnected the microphone and amplifier unit pneumatically and allowed for SPEQ. The FCB cable was wrapped around the silicone tube and then fed into the amplifier housing. A separation distance of 100 mm between the microphone and amplifier ensured minimal interference with the surgical access window and minimal electromagnetic interference with the microphone output signal.

In the final step of ICAR fabrication, the SR tube was inserted into the guiding pin of the base plate and secured using UV curable acrylate. This adhesive provided a sufficiently strong yet releasable bond, which allowed the simple replacement of the SR if broken during testing.

### 2.4. Functional Tests and Performance under Laboratory Conditions

Functional tests of the ICAR were performed in water using a calibration technique for hydrophones that was developed by Schloss et al. [22]. The method is based on a vibrating water column that is driven by a shaker (Type 4810, Brüel&Kjær Sound & Vibration Measurement A/S, Nærum, Denmark). The ICAR under investigation was immersed in the water column at a controlled depth. The pressure variation at the controlled immersion depth was caused by variation of the hydrostatic head and by the inertia of the water column above the ICAR head.

To determine the equivalent input noise (EIN) level, the vibration generator was switched off and the output signal of the ICAR in the water column was recorded. The frequency response and EIN of the ICAR were measured for frequencies between 0.25 and 8 kHz. Details of this setup are reported in a separate publication [6].

### 2.5. Sensor Validation: ICAR Use in Acute Animal Experiments

#### 2.5.1. Surgical Approach

The similarities of the middle and inner ear anatomy and physiology provided the rationale for selection of sheep as a valid model to conduct testing of implanted hearing devices for humans [16,18,20,21]. The front part of the scala tympani, beginning approximately 2–3 mm from the round window, is the largest cavity of the sheep cochlea and, hence, is best suited for ICAR placement. To reach this part of the cochlea, the surgical approaches reported in the literature [15,16] were adapted by further development and training regarding surgical access to the cochlea and ICAR placement in sheep cadavers. All preparation and validation experiments were performed with adult Swiss white-alpine sheep.

Acute experiments were conducted according to the Swiss regulations of Animal Welfare. The local federal authorities (code ZH 136/16) granted permission. Sheep were held, housed and fed according to Swiss legislation for experimental animals and fasted for 24 h before induction of anaesthesia, but access to water was granted at all times. Anaesthetization was performed according to institutional and approved standards. Sufficient analgesia was provided and anaesthesia and analgesia were monitored adopting the highest possible standards of animal care.

**Figure 2:**
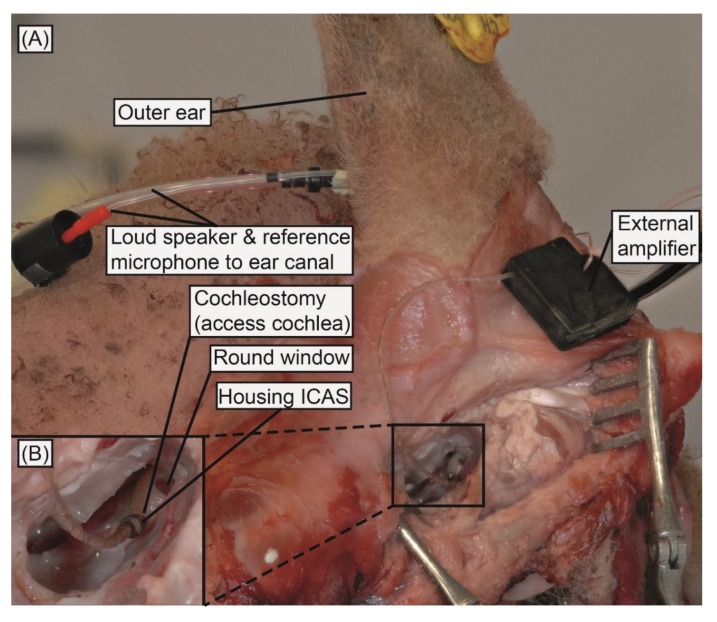
(**A**) ICAR inserted into the sheep cochlea (preparation experiment on cadaver head). (**B**) Enlarged view of inserted ICAR.

The lateral side of the sheep’s head was shaved and a skin incision was made. The temporal bone was exposed and bleeding controlled. The bulla was opened and the bony otic capsule containing the basal part of the cochlea was exposed. An access (cochleostomy) was drilled into the scala tympani using a 0.7 mm diamond burr at low speed (position inferior and slightly anterior to the round window membrane). First, the bone was thinned out before the opening of the remaining thin bony layer and the endost of the cochlea were conducted under water to ensure that no air entered the cochleostomy. For the ICAR insertion, the amplifier unit was first fixed with a bone screw in the mastoid. Next, the ICAR was inserted into the scala tympani through the cochleostomy using forceps. The ICAR was inserted until the base of the microphone housing, sealed with fatty tissue, and secured (Figure 2). The corresponding insertion depth of the SR in the scala tympani was approximately 1 mm. The surgeon’s subjective satisfaction with the ICAR was validated concerning ease of use and satisfaction with the system design.

#### 2.5.2. ICSP Measurements in Acute Animal Experiments

To improve the validity of the animal experiments, the ICSP measurements were repeated twice using two ICARs implanted serially into one animal. The probe tube of a loudspeaker (ER-2, Etymotic Research, Elk Grove Village, IL, USA) and a reference microphone (ER-7C, Etymotic Research, USA) were inserted with a foam insert earphone into the ear canal of the sheep. Stepped sinusoidal acoustic stimuli in the frequency range of 0.25–8 kHz were generated by an audio analyzer (APx585, Audio Precision Inc., Beaverton, OR, USA) and delivered to the loudspeaker via an audio amplifier (RMX 850, QSC Audio Products, Costa Mesa, CA, USA). Twenty stimulus frequencies were distributed logarithmically within the considered frequency range. A sound pressure level of >90 dB SPL in the ear canal was maintained across the stimulus frequencies. Placement of the foam insert proximate to the tympanic membrane was not achieved due to the “angularly shaped” anatomy of the sheep’s ear canal. The remaining cavity caused destructive interference in the sound pressure field within the duct system of the reference microphone and the loud speaker at a frequency of 5 kHz. This abrupt drop in sound pressure was identified on the reference microphone but not on the ICSP signal. Consequently, the reference microphone signal in that frequency range (4–5.5 kHz) was regarded as an unreliable interpretation of the SPL acting on the tympanic membrane and was corrected based on earlier investigations on sheep cadavers [18].

The ICAR and the reference microphone signal were simultaneously recorded with two analog input channels of an audio analyzer (APx585, Audio Precision Inc., Beaverton, OR, USA). The waveform of each stimulation frequency was sampled at 100 kHz for 0.2 s. A custom LabVIEW (Version 2013 SR1, National Instrument, Austin, TX, USA) application was used to generate and record the analog signals. The raw data were post-processed by a 1/3-octave band-pass filter (digital third-order Butterworth filter) with center frequencies corresponding to the acoustic stimuli. The RMS value of the band-pass filtered signal was averaged over five subsequent measurements in order to reduce the noise floor of the measurements. Data analysis and illustration were completed using MATLAB 2017a (MathWorks Inc., Natick, MA, USA) and GraphPad Prism V5.04 (GraphPad Software Inc., La Jolla, CA, USA) software, respectively.

Prior to ICSP measurement, the 1/3-octave EIN level of the inserted ICAR was recorded to verify the ICAR’s condition after surgical insertion. The acoustic stimulation was then switched off and the output signal of the transducer acquired while the stepped frequency sweep/ filtering was performed by the LabVIEW application.

## 3. Results

### 3.1. Functional Tests and Performance under Laboratory Conditions

**Figure 3:**
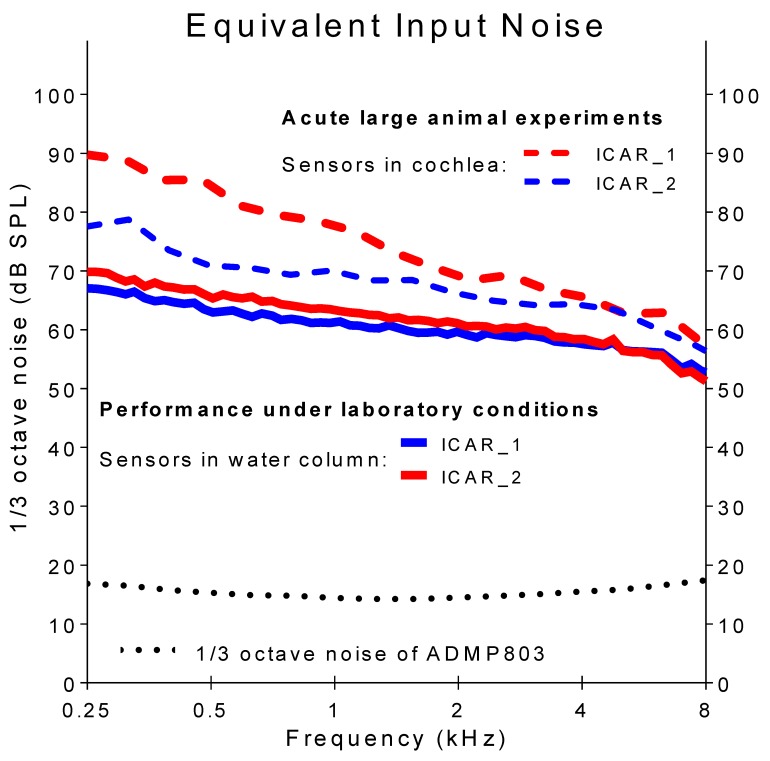
Equivalent input noise (EIN) of the ICARs under laboratory conditions (solid lines) and during acute large animal experiments (dashed lines).

Figure 3 displays the 1/3-octave EIN levels (solid lines) of the two ICARs used for the in-vivo experiments in both laboratory and in-vivo conditions. The data were gathered under laboratory conditions using the vibrating water column setup. The EIN level decayed with increasing frequency from approximately 70 dB SPL at 0.25 kHz to 50 dB SPL at the maximum considered frequency of 8 kHz. For comparison, the noise level of the MEMS CMIC without the PD (ADMP803 [25], black dotted line) was situated between 15 and 17 dB SPL across the whole frequency range. The higher EIN of the ICAR resulted from the PD, which added additional mechanical stiffness to the receiver and thereby reduced its sensitivity. Details of this ICAR behavior are reported in a separate publication with similar PD’s [6]. The polyimide diaphragm currently used shows compressive intrinsic stress, which caused diaphragm buckling and thus enhanced stiffness compared with a flat diaphragm with zero or minimal tensile stresses.

### 3.2. Animal Experiments

#### 3.2.1. Surgical Approach

Two surgeons (A.H., D.P.) performed the ICAR insertion into the cochlea using forceps, and both reported convenient handling of the ICAR during insertion. The size and geometry of the ICAR housing enabled simple placement in the middle ear cavity and a permanent view of the SR head during ICAR insertion through a cochleostomy (ICAR navigation). Neither the microphone housing nor the silicon tubing connecting the microphone and the amplifier unit were secured after ICAR placement. Movement of the ICAR due to extensive stresses within the flexible interface was not observed. The robustness of the ICAR was confirmed by several insertions without seeing damage or changes in sensing sensitivity.

#### 3.2.2. ICSP Measurements in Acute Animal Experiments

During the animal experiments, the noise level of both ICARs was higher than under laboratory conditions (Figure 3, dashed lines ICAR_1 and ICAR_2). In particular, ICAR_2 showed 20 dB higher EIN in the low frequencies compared to the measurements in the laboratory. It is assumed that acoustic and electromagnetic interference with various hardware (e.g., the surgical microscope, instruments to monitor vital functions, surgical table) used in the operating theatre and the higher ambient temperature (the water in the calibration setup was 21 °C) in a living subject (sheep body core temperature is >37 °C) caused the increased noise at low frequencies.

**Figure 4:**
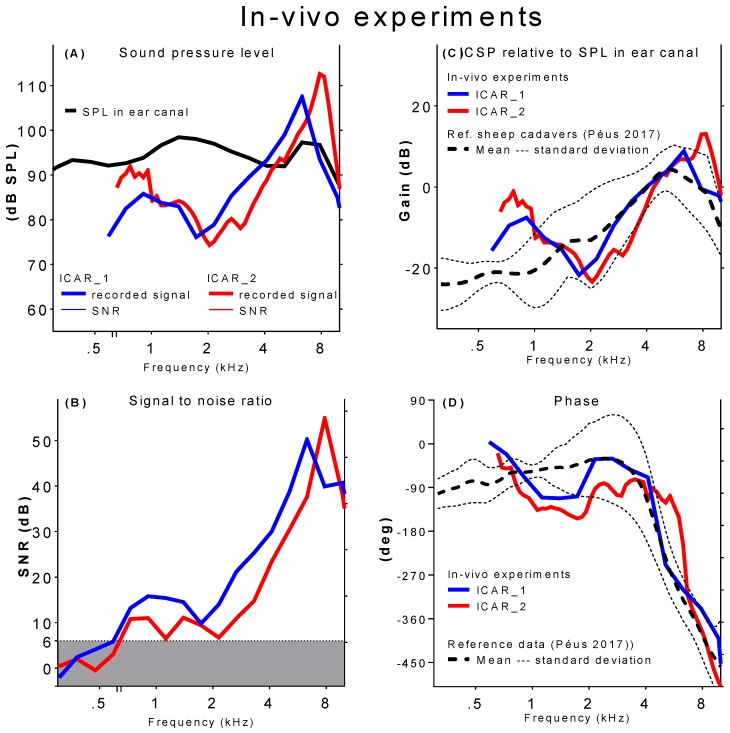
Acute sheep experiments (ICAR _1 blue lines; ICAR_2 red lines): (**A**) Intracochlear sound pressure level (solid lines) and corresponding sound pressure level in the ear canal (dashed line). (**B**) SNR (solid lines) shown as difference between ICSP (**A**) and noise measurements (dashed lines in Figure 3). The grey shaded area represents SNR < 6 dB. Recorded ICSP normalized by the measured sound pressure in the ear canal: magnitude (**C**) and phase (**D**).

Figure 4 summarizes preliminary results from ICSP measurements conducted during the in vivo experiments. The sound pressure in the basal part of the scala tympani as a function of the frequency is shown in Figure 4A (solid lines). The sound pressure in the ear canal measured using the reference microphone is indicated by the black dashed line. Acoustic stimulation between 89 and 100 dB SPL was maintained over the considered frequency range. Displayed measurement points are averages over five subsequent measurements at the same stimulation frequency. The standard deviation in the five repetitive recordings was below 0.8dB for all measured frequency points.

Only ICSP data with SNRs higher than 6 dB were considered valid. Pressure data below 620 Hz (ICAR_1) and 650 Hz (ICAR_2), respectively, were excluded from the graphical representation (cf., Figure 4B). Peaks in ICSP were identified at 6.5 kHz (ICAR_1) and 8 kHz (ICAR_2). Despite the flat frequency response acoustic stimulation, the ICSP decayed rapidly for frequencies lower and higher than the corresponding peak frequency. For frequencies lower than 2 kHz, the ICSP again became greater.

The ICSP data from in vivo experiments were compared with reference data obtained from tests in sheep cadaver ([18] (cf., Figure 4 (C&D)). The upper figure displays the magnitude of the ICSP normalized with the corresponding pressure in the ear canal. The resulting ratio is indicated as a gain. The reference data represent the mean and standard deviation of experiments in six sheep cadaver heads (dashed and dotted black lines). The corresponding phase response (i.e., phase shift between stimulation and ICSP) is depicted in Figure 4D. The phase change was about 400 degrees over the frequency range 0.6–8 kHz with more rapid phase changes near the resonance frequency.

A satisfactory agreement between experiments in vivo and in cadavers existed for frequencies above 2 kHz. The characteristic shape of the middle ear transfer function in sheep with a pronounced peak value appearing between 5 and 8 kHz was clearly reflected in the in-vivo experiments. At lower frequencies, both the gain and the phase deviated from the reference data.

## 4. Discussion and Conclusions

The goal of this work was to demonstrate the feasibility and validation of an ICAR designed to measure ICSP in acute large animal experiments. The obtained results are promising; the main requirements defined to accomplish this task, such as an appropriate form factor of the ICAR system and sufficiently high sensing performance, were fulfilled. Surgeons who performed the ICAR insertion into the cochlea reported ease of use and satisfaction with the system design.

It is important to point out the difference between testing under laboratory conditions and during acute experiments in the operating theatre. The time for acute experiments is limited by the maximal duration of anesthesia, the restricted positioning and access of the sheep head compared to cadaver heads, and management of hemostasis. Interference with acoustic and electrical noise sources during ICSP measurement in the operating theatre have been identified as a challenge compared with a controlled isolated measurement setup under laboratory conditions. However, the ICAR experiments were successfully performed in both conditions, and we gained important procedural knowledge for future in vivo (acute and chronic) experiments.

The consequence of an elevated noise level of the ICAR due to the interference with acoustic and electrical noise sources during the in-vivo experiment was a low SNR, especially within the lower frequency range where discrepancies between the in vivo experiment and reference data occurred. Several improvements could be implemented to increase the SNR of future experiments: (1) Residual stresses in the polyimide PD caused buckling of the diaphragm and thereby lowered the membrane’s compliance thus affecting the mechanical sensitivity of the ICAR. Optimization of the fabrication process for polyimide PDs could lead to lower residual stresses and, therefore, to improved sensing performance of the ICAR; (2) The study ICAR had only one PD at the SR tip. A multi-diaphragm SR design with an increased overall sensing area would provide higher ICAR sensitivity and a better SNR. (3) Maximum acoustic stimulation levels of only 100 dB SPL were applied during the experiments. Higher stimulation levels (e.g., 130 dB SPL in [2,3]) would lead to higher ICSP and therefore higher SNR. However, such a high stimulation requires a sound source that is located very close to the tympanic membrane. Because of the narrow and curved ear canal of the sheep, appropriate installation of a device for acoustic stimulation is challenging and needs to be further explored. (4) Improved shielding of the ICAR could minimize electrical interferences and therefore, lower the noise level.

Several studies [2,3,4] have shown that the tolerance range for experimental ICSP measurements is large (>10 dB). The ICSP measurements presented in this paper yielded comparable results to findings in sheep temporal bones [18] and confirmed the main aim, which was to demonstrate the feasibility of ICSP measurements in acute sheep experiments. Using an optimized version of the study ICAR to further explore the mechanisms that lead to differences between in-vivo and cadaver data representing the biomechanics of hearing (post-mortem effects and conservation techniques) will be a goal for future experiments.

Above all, the findings from the present study shall be incorporated into the next generation ICAR, which is being developed for use in chronic sheep experiments. Such an application demands that additional design requirements are met, including higher sensing performance, biocompatible and hermetic packaging as well as parts for system integration (e.g., a percutaneous plug). High sensing performance and simplicity of system integration are important criteria when considering use of the ICAR concept as a microphone integrated into a CI electrode for a totally implantable cochlear implant system.
